# Evaluation of Hepatic/Renal and Splenic/Renal Echointensity Ratio Using Ultrasonography in Diabetic Nephropathy

**DOI:** 10.3390/diagnostics13142401

**Published:** 2023-07-18

**Authors:** Melike Elif Kalfaoglu

**Affiliations:** Department of Radiology, Abant Izzet Baysal University Hospital, Bolu 14280, Turkey; melikekalfaoglu@hotmail.com

**Keywords:** diabetic nephropathy, ultrasonography, echointensity, type 2 diabetes mellitus

## Abstract

The objective of this study is to assess the hepatic/renal and splenic/renal echointensity ratios in ultrasonography in patients with and without diabetic nephropathy. This retrospective study included patients with diabetes mellitus who underwent ultrasound examinations at our hospital between January 2023 and May 2023. Ultrasound examinations were conducted using renal cortical echogenicity and corticomedullary differentiation by using B-mode ultrasonography. The hepatic/renal and splenic/renal echo intensity ratios were compared among study groups (diabetic patients with diabetic nephropathy and without nephropathy). The diabetic nephropathy group exhibited significantly higher right renal echointensity and left renal echointensity compared to the non-nephropathic group. Additionally, the splenic/renal echointensity ratio and hepatic/renal echointensity ratio were significantly lower in the diabetic nephropathy group. Urinary microalbumin levels were significantly correlated with right renal echointensity (r = 0.65, *p* < 0.001) and left renal echointensity (r = 0.69, *p* < 0.001). There was also a significant inverse correlation between the urinary albumin and splenic/renal echointensity ratio (r = −0.58, *p* < 0.001). Ultrasonography, specifically the assessment of hepatic/renal and splenic/renal echointensity ratios, shows promise as a noninvasive and cost-effective method for evaluating morphological changes in the kidneys in patients with diabetic nephropathy. These findings suggest that ultrasonography can be a valuable tool for monitoring the progression of diabetic nephropathy and contributing to its early detection and management.

## 1. Introduction

Diabetes mellitus (DM) is a group of metabolic diseases characterized by hyperglycemia, which refers to high levels of glucose in the blood. These conditions arise from defects in insulin secretion, insulin action, or both. The chronic hyperglycemia associated with diabetes can lead to long-term damage, dysfunction, and failure of various organs, including the eyes, kidneys, nerves, heart, and blood vessels.

Multiple pathogenic processes contribute to the development of diabetes. These processes range from autoimmune destruction of pancreatic beta cells, resulting in insulin deficiency, to abnormalities that lead to insulin resistance. The underlying problem in diabetes is the inadequate effect of insulin on target tissues, which impairs carbohydrate, fat, and protein metabolism. Insufficient insulin secretion and diminished tissue responses to insulin can coexist in the same patient, making it often unclear which abnormality, if any, is the primary cause of hyperglycemia [1]. According to the latest data provided by the International Diabetes Federation, there are approximately 415 million adults aged 20 to 79 worldwide affected by diabetes. This number is projected to increase to 642 million by the year 2040, with the prevalence rising from 8.8% to 10.4%. Despite the high prevalence of diagnosed diabetes, a significant portion of the population remains unaware of their condition, representing nearly half of all people with diabetes.

When considering different regions, the age-adjusted prevalence of diabetes is as follows: 3.8% in Africa, 7.3% in Europe, 10.7% in the Middle East and North Africa, 11.5% in North America and the Caribbean, 9.6% in South and Central America, 9.1% in Southeast Asia, and 8.8% in the Western Pacific region. China, India, and the United States are the top three countries with the highest number of individuals affected by diabetes [2].

By 2019, diabetes accounted for 1.5 million deaths directly, with 48% of these deaths occurring before the age of 70. Additionally, diabetes was responsible for around 460,000 deaths related to kidney disease. Raised blood glucose levels contributed to approximately 20% of deaths caused by cardiovascular diseases [3]. DM has three common types: type 1, type 2, and gestational diabetes. The most prevalent type is type 2 DM, which accounts for approximately 90–95% of all diagnosed cases of DM. Other types of diabetes such as maturity-onset diabetes of youth or latent autoimmune diabetes in adults, among others, are caused by specific genetic conditions or from surgery, medications, infections, pancreatic disease, or other illnesses. Such types of diabetes account for 1% to 5% of all diagnosed cases [4]. Due to the slow development of symptoms, mild progression in the initial years, and the unclear relationship with DM, the diagnosis of type 2 DM can be delayed. It has been shown that individuals with impaired fasting plasma glucose tests can develop type 2 diabetes up to 8 years after the initial blood tests, indicating the presence of a long prodromal phase in patients with type 2 DM [5].

Diabetic nephropathy (DN) is a common and severe complication of diabetes mellitus (DM) and is linked to higher morbidity and mortality rates in diabetic individuals [6]. The prevalence of diabetic nephropathy varies among different races and ethnic groups. In the United States, the number of diabetic patients commencing treatment for end-stage renal disease (ESRD) has shown a significant rise, going from over 40,000 in 2000 to over 50,000 in 2014 [7]. In China, there has also been a substantial increase in the incidence and prevalence of DN over the past decade. The estimated number of diabetic patients with chronic kidney disease (CKD) in China has reached 24.3 million [8]. On a global scale, the occurrence of diabetes is swiftly on the rise, especially in developing countries [9]. Unless prompt enhancements are made in the clinical approaches aimed at preventing DN, the frequency of DN is expected to increase proportionally with the expanding prevalence of diabetes [10].

Diabetic nephropathy is a renal complication caused by microvascular complications of diabetes, particularly prevalent in individuals with type 2 diabetes mellitus. It stands as the leading cause of end-stage renal disease worldwide [11]. It develops in approximately 40% of patients with diabetes, after 10 years of type 2 diabetes mellitus were diagnosed [12]. Diabetic nephropathy is a diagnosis that refers to specific pathological structural and functional changes in the kidneys of individuals with diabetes mellitus (DM). In diabetic nephropathy, there is an occurrence of proteinuria, an increase in serum creatinine concentration, and a decrease in the glomerular filtration rate (GFR). The process begins with proteinuria as a result of various pathophysiological mechanisms caused by DM, eventually leading to progressive impairment of kidney functions and chronic kidney failure [13]. It is crucial to identify individuals who are more susceptible to developing diabetic nephropathy in order to better manage the disease progression. Numerous factors and mechanisms contribute to the development and outcome of diabetic nephropathy. Albuminuria has been widely used as a biomarker to assess renal function and is typically associated with glomerular injury and increased permeability to macromolecules [14,15]. However, it may not be detectable in the early stages of the disease. Despite its common usage, albuminuria has several limitations, including high variability and low sensitivity. It cannot reliably predict renal outcomes and is not specific to diabetic nephropathy [16].

The diagnosis of diabetic nephropathy is based on medical history, physical examination, and laboratory tests. Kidney imaging methods such as ultrasound are typically performed to rule out other kidney and urinary tract pathologies. Ultrasonography serves as a noninvasive and cost-effective diagnostic tool, offering comprehensive anatomical information essential for the diagnosis of renal diseases, while avoiding the use of radiation or contrast agents. Consequently, it has globally replaced conventional radiography [17,18]. These factors collectively facilitate the early identification and prediction of abnormal renal function tests, crucial for determining appropriate therapeutic interventions. However, ultrasound findings in diabetic nephropathy are often nonspecific and can be normal. In diabetic nephropathy or chronic kidney disease due to other causes, typical ultrasound findings include decreased kidney length and renal cortical thickness, increased renal cortical echogenicity, decreased differentiation of renal pyramids and sinuses, and irregular contours with papillary calcifications [19,20]. These details assist in identifying the extent of renal parenchymal damage and the possibility of its reversibility [21], and the decision to perform a renal biopsy [22].

In ultrasonography, renal cortex echogenicity is evaluated in relation to the echogenicity of neighboring liver, spleen, and renal sinuses. Spleen echogenicity is used as a standard reference to evaluate the echogenicity of the renal cortex in the presence of fatty liver [23]. In the early stages of diabetes, kidney size increases due to hyperfiltration, while in later stages, kidney size decreases due to glomerulosclerosis. Additionally, an increase in renal cortical echogenicity suggests a deterioration in kidney function. Ultrasonography is also used to exclude nondiabetes-related kidney disorders such as kidney stones, masses, or hydronephrosis [24,25].

According to the original article, it has been reported that the best sonographic parameter correlating with serum creatinine in chronic kidney disease is renal cortical echogenicity and its grading in comparison to longitudinal length, parenchymal thickness, and cortical thickness [26]. As a result of the presence of collagen, the echogenicity is heightened in cases of interstitial fibrosis and glomerulosclerosis, although this has not been widely acknowledged [27].

The increase in echogenicity may also contribute to interstitial inflammation. While the human eye can subjectively evaluate echogenicity, it is considered unreliable [28]. The echogenicity parameter of the cortex, which indicates changes in renal cortical structure, is important; however, echogenicity is currently measured qualitatively. Therefore, Manley et al. have developed a qualitative method for measuring renal cortical echogenicity [29].

There are studies in the literature indicating that ultrasonographic imaging can contribute to the evaluation of morphological changes in the kidneys in diabetic nephropathy and assist in monitoring the progression of the disease [30]. Additionally, there are studies suggesting that Doppler Ultrasonography can provide valuable insights in detecting early changes in asymptomatic nephropathy [31]. Zang et al. reported that quantitative assessment of the hepatic/renal echo intensity ratio using ultrasonography is an easy and effective parameter for the early diagnosis of mild hepatic steatosis and evaluation of the effectiveness of nonalcoholic fatty liver disease treatment [32].

However, to the best of our knowledge, the evaluation of the hepatic/renal–splenic/renal echo intensity ratio has not been previously investigated in diabetic nephropathy. Therefore, in our study, we aimed to evaluate and compare the echo intensity values with laboratory data in patients with and without diabetic nephropathy.

## 2. Materials and Methods

The present retrospective study was conducted in the radiology department of Abant Izzet Baysal University Hospital after obtaining ethical approval (2023/156).

The demographic information (age, gender, comorbidities, etc.), laboratory data (glucose, HbA1C, blood urea nitrogen, estimated glomerular filtration rate, etc.), and recorded ultrasound reports and images of patients who visited our hospital between 1 January 2023 and 20 May 2023 for follow-up related to diabetes mellitus (DM) were retrospectively scanned from our hospital’s PACS system. Two groups were created: patients with diabetic nephropathy and patients without diabetic nephropathy. Patients with incomplete or inadequate investigations, suboptimal ultrasound examinations, a history of surgery, or a history of malignancy were excluded from the study. After applying the exclusion criteria, a total of 59 cases were included in the study. All examinations were performed using a Samsung RS 85 prestige ultrasound machine (Samsung Electronics, Suwon, South Korea) with a curved array transducer (CA1-7A; 1–7 MHz). A curved array transducer with a frequency range of 1–7 MHz was used for the ultrasound examination. The ultrasound examinations were performed by a single radiologist with 20 years of experience, who was blinded to the patients’ clinical details and laboratory findings. During the measurement, the probe frequency was set at 4.2 MHz and the depth was maintained at 16 cm. The gain was set at 52 dB and the dynamic range was set at 55 dB for all measurements, with these settings kept consistent throughout. The Time–Gain Control was maintained in a neutral position for each patient. Renal cortical echogenicity and cortico-medullary differentiation were evaluated using B-mode ultrasonography. Renal cortical echogenicity was compared and graded in relation to the echogenicity of the liver and spleen, as well as the renal medulla. The grading system was as follows: Grade 0: Normal echogenicity, which is less than that of the liver, with maintained corticomedullary differentiation. Grade 1: Echogenicity similar to that of the liver, with maintained corticomedullary differentiation. Grade 2: Echogenicity greater than that of the liver, with maintained corticomedullary differentiation. Grade 3: Echogenicity greater than that of the liver, with poorly maintained corticomedullary differentiation. Grade 4: Echogenicity greater than that of the liver, with a loss of corticomedullary differentiation. The liver echogenicity was evaluated in B-mode ultrasonography based on several US features, including liver brightness, contrast between the liver and the kidney, US appearance of the intrahepatic vessels, liver parenchyma, and diaphragm. Steatosis was graded according to the following criteria: absent (normal) when the liver echotexture appeared normal; mild (grade 1) when there was a slight and diffuse increase in liver echogenicity with normal visualization of the diaphragm and the portal vein wall; moderate (grade 2) when there was a moderate increase in liver echogenicity with slightly impaired appearance of the portal vein wall and the diaphragm; severe (grade 3) when there was a marked increase in liver echogenicity with poor or no visualization of the portal vein wall, diaphragm, and posterior part of the right liver lobe.

**Figure 1:**
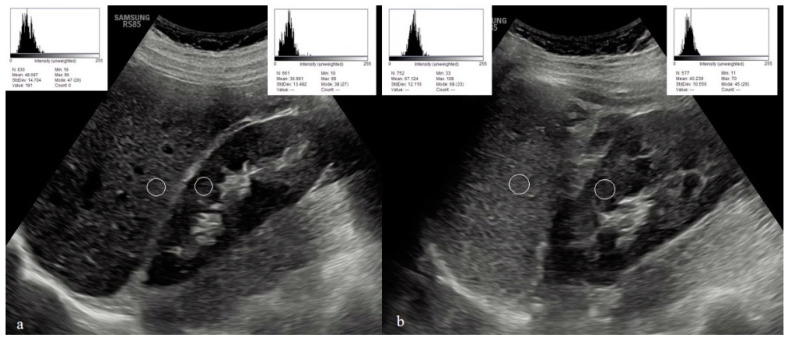
Hepatic/renal (**a**), and splenic/renal (**b**) echointensity measurements. The white circles correspond to the regions of interest used in the ultrasound analyses.

To evaluate the US hepatic/renal (HR) echointensity ratio and spleen/renal (SR) echointensity ratio, ultrasound images with clear visualization of the liver, right kidney, spleen, and left kidney were obtained in the sagittal liver/right kidney view with the patient in the left lateral position, and in the sagittal spleen/left kidney view with the patient in the right lateral position. The obtained ultrasound images were transferred to a computer system. The images were evaluated using Image-J (Java 1.8.0_172 (64-bit), Wayne Rasband and contributors National Institutes of Health, USA) program. The regions of interest (ROIs) for the hepatic and right renal areas, as well as the splenic and left renal areas, were selected at the same depth along the focusing area near the center of the image to minimize image distortion effects and beam attenuation. In the liver parenchyma, an ROI was chosen excluding blood vessels, bile ducts, or focal liver lesions. In the spleen parenchyma, an ROI was chosen excluding blood vessels. In both renal cortices, an ROI was selected excluding large vessels, renal sinus, masses, or cysts. Any regions affected by artifacts were also excluded. In the program, the black and white pixels in the images obtained from the ultrasound examination were assigned values ranging from 0 to 255, and a histogram curve was generated. Higher echointensity values indicated poorer renal parenchymal echogenicity, while lower echointensity values indicated better renal parenchymal echogenicity. The sonographic hepatic/renal (HR) ratio was calculated by dividing the mean echo intensity of the pixels within the selected hepatic ROI by the mean echo intensity of the pixels within the selected ROIs of the right renal cortex. Similarly, the sonographic spleen/renal (SR) ratio was calculated by dividing the mean echo intensity of the pixels within the selected splenic ROI by the mean echo intensity of the pixels within the selected ROIs of the left renal cortex. Figure 1 shows hepatic/renal and splenic renal echointensity measurements.

### Statistical Analyses

Statistical software (SPSS 18 for Windows, IBM Co, Chicago, IL, USA) was used for statistical analyses. Kolmogorov Smirnov test was applied to the study variables for normality analysis. Variables that fit into normal distribution were conducted using independent samples *t* test and expressed as means and standard deviations. Other variables that do not fit into normal distribution were expressed as median (min–max) and compared with the Mann–Whitney U test. Categorical variables were compared between study groups with chi-square test and given as numbers and percentages. Correlation between study variables was analyzed using Pearson’s correlation test. Specificity and sensitivity of study parameters in detecting nephropathy were analyzed using the ROC analysis test. It is considered as significant when the *p* value was lower than 5%.

## 3. Results

A total of 59 subjects were enrolled in the present work after the exclusion of subjects according to the exclusion criteria. Of those 35 were in the non-nephropatic group and 24 were in the diabetic nephropathy group. Mean ages of the non-nephropatic and diabetic nephropathy groups were 58.5 ± 12 years and 61.7 ± 10 years, respectively (*p* = 0.29). There were 10 (42%) women and 14 (58%) men in the diabetic nephropathy group, while there were 20 (57%) women and 15 (43%) men in non-nephropatic subjects (*p* = 0.24), (Table 1).

The mean albumin level of the diabetic nephropathy group was 4.6 ± 0.4 g/dL and the albumin of non-nephropathic subjects was 4.7 ± 0.4 g/dL (*p* = 0.58).

The median AST level of the diabetic nephropathy group was 18 (10–46) U/L and the AST of non-nephropathic subjects was 18 (10–41) U/L (*p* = 0.91).

The median ALT level of the diabetic nephropathy group was 21 (11–58) U/L and the ALT of non-nephropathic patients was 18 (10–49) U/L (*p* = 0.62).

The median urea level of the diabetic nephropathy group was 35 (12–92) mg/dL and the urea of non-nephropathic subjects was 35 (23–58) mg/dL (*p* = 0.88).

The median eGFR level of the diabetic nephropathy group was 81 (32–110) ml/min/1.73 m^2^ and the eGFR of non-nephropathic patients was 90 (55–11) ml/min/1.73 m^2^ (*p* = 0.13).

The mean right renal widths of the diabetic nephropathy and non-nephropatic groups were 45 ± 5.5 mm and 44 ± 5.4 mm, respectively (*p* = 0.58).

The mean left renal widths of the diabetic nephropathy and non-nephropatic groups were 51 ± 6.7 mm and 49 ± 6.2 mm, respectively (*p* = 0.17).

There were no statistical differences between the diabetic nephropathy group and non-nephropathic subjects in terms of liver length (*p* = 0.85), left renal length (*p* = 0.42), and spleen length (*p* = 0.27).

The median right renal cortical thickness of the diabetic nephropathy group was 15 (11–18) mm and the right renal cortical thickness of the non-nephropathic subjects was 14 (8–18) mm (*p* = 0.06).

There were no statistical differences between the diabetic nephropathy group and non-nephropathic subjects in terms of liver echogenicity (*p* = 0.29), right renal echogenicity (*p* = 0.17), and left renal echogenicity (*p* = 0.06).

There were no statistical differences between the diabetic nephropathy group and non-nephropathic patients in terms of liver echointensity (*p* = 0.40) and spleen echointensity (*p* = 0.62).

The mean BMI was significantly higher in the diabetic nephropathy group (32.4 ± 1) kg/m^2^ compared to that in the non-nephropathic group (29.1 ± 0.7 kg/ m^2^), (*p* = 0.02).

Median microalbumin in urine was significantly higher in the diabetic nephropathy group 122.5 (33–2000) mcg/mL compared to that in the non-nephropathic group 5 (4–18) mcg/mL, (*p* < 0.001).

The median serum glucose level was higher in the diabetic nephropathy group 158 (89–432) mg/dL compared to that in the non-nephropathic group 137 (73–313) mg/dL (*p* = 0.046). Similarly, the median HbA1C of the diabetic nephropathy group (8.5% (7.1–12.6%)) was significantly higher than that of the non-nephropathic group (7.2% (5.2–12.2%)) (*p* < 0.001).

There was also a significantly higher median serum creatinine level in the diabetic nephropathy group (0.92 (0.58–9) mg/dL) compared to the non-nephropathic group (0.80 (0.51–1.31) mg/dL) (*p* = 0.01).

The mean splenic/renal echointensity ratio was significantly lower in the diabetic nephropathy group (0.60 ± 0.2)% compared to that in the non-nephropathic group (1.02 ± 0.2)%, (*p* < 0.001).

The median right renal echointensity was higher in the diabetic nephropathy group (59.6 (44.6–80.8)) compared to that in the non-nephropathic group (32.7 (28.7–52)), (*p* < 0.001). Similarly, the median left renal echointensity of the diabetic nephropathy group 58 (41.4–83.5) was significantly higher than that of the non-nephropathic group (33.9 (29.1–58.7)), (*p* < 0.001).

The median mean splenic/renal echointensity ratio was significantly lower in the diabetic nephropathy subjects (1.43 (0.44–2.2))% compared to that in the non-nephropathic patients (1.9 (1.05–3.3))%, (*p* = 0.025).

Additionally, although within normal limits, there were differences in the left renal cortical thickness (*p* = 0.006) and right renal length (*p* = 0.04) between the diabetic nephropathy group and non-nephropathic subjects.

Table 2 summarizes the data of diabetic nephropathy and non-nephropathic groups.

In the Pearson’s correlation analysis, the urinary microalbumin levels were significantly correlated with right renal echointensity (r = 0.65, *p* < 0.001) and left renal echointensity (r = 0.69, *p* < 0.001). There was also a significant inverse correlation between the urinary albumin and splenic/renal echointensity ratio (r = −0.58, *p* < 0.001).

In ROC analysis the sensitivity and specificity of right renal echointensity (when higher than 44.15) in detecting diabetic nephropathy were 100% and 97%, respectively (AUC: 0.99, *p* < 0.001, 95% CI: 0.98–1). Similarly, in ROC analysis, the sensitivity and specificity of left renal echointensity (when higher than 39.18) in detecting diabetic nephropathy were 100% and 91%, respectively (AUC: 0.98, *p* < 0.001, 95% CI: 0.95–1).

According to the results of the ROC analysis, the sensitivity and specificity of the hepatic/renal echointensity ratio (when lower than 1.9) in detecting diabetic nephropathy were 87% and 51%, respectively (AUC: 0.67, *p* = 0.03, 95% CI: 0.53–0.81). Similarly, the sensitivity and specificity of the splenic/renal echointensity ratio (when lower than 1.2) in detecting diabetic nephropathy were 100% and 97%, respectively (AUC: 0.98, *p* < 0.001, 95% CI: 0.94–1). Table 3 summarizes the ROC analysis results in diabetic nephropathy groups.

## 4. Discussion

The present study provides compelling evidence supporting the utilization of renal echointensity, the hepatic/renal echointensity ratio, and the splenic/renal echointensity ratio as reliable markers for diabetic nephropathy. These findings strongly suggest that alterations in renal echointensity and the ratios of hepatic and splenic echointensity to renal echointensity possess significant diagnostic and prognostic value in assessing the presence and progression of diabetic nephropathy. The observed changes in renal echointensity were specific to the kidneys, with no corresponding alterations in splenic and hepatic echointensity. The significant correlation between renal echointensity values and urinary microalbumin levels, along with the inverse correlation between the urinary albumin and splenic/renal echointensity ratio, supports the notion that renal echointensity reflects structural changes in the renal parenchyma, including glomerular and tubular alterations, fibrosis, and inflammation. The increased renal echointensity observed in the diabetic nephropathy group suggests the presence of such structural changes, contributing to the progressive deterioration in renal function.

Consistent with the existing literature, diffuse renal parenchymal diseases are known to exhibit increased parenchymal echogenicity on gray scale ultrasound [33]. Moreover, previous studies have reported the direct influence of diabetes on kidney morphology, with early-stage manifestations of renal volume enlargement and cortical thickening followed by late-stage atrophy and increased echogenicity [30]. Xia et al. have demonstrated the potential of using the ultrasound hepatic/renal ratio and hepatic attenuation rate to quantify liver fat content noninvasively [34]. Additionally, ultrasound evaluation of lower limb muscles and echointensity assessment have emerged as useful approaches for identifying sarcopenia [35]. Although our study did not find statistically significant differences in renal echogenicity between the diabetic nephropathy and non-nephropathic groups, the significant disparities in renal echointensity values indicate the potential of quantitative assessment in detecting subtle structural changes that may not be captured by qualitative evaluation alone. This highlights the importance of quantitative evaluation as an objective and reliable measure of renal echointensity, surpassing the limitations of qualitative assessment, which is prone to subjectivity and interoperator variability. Emphasizing the advantages of the quantitative approach, our study provides valuable insights into the extent of renal damage and serves as an indispensable tool for assessing the progression of the disease. These findings underscore the significance of incorporating quantitative methods for a comprehensive characterization of renal echointensity changes in diabetic nephropathy, contributing to our understanding of the disease’s pathophysiology and aiding in the development of more accurate diagnostic and monitoring strategies.

According to genetic evidence, there is a causal association between higher BMI levels and an increased risk of diabetic nephropathy and decreased estimated glomerular filtration rate (eGFR) levels [36]. Interestingly, this association appears to be more pronounced in women, as the increase in BMI level has a greater impact on DN risk in women compared to men. Furthermore, among individuals with type 2 diabetes mellitus (T2DM), both central and general obesity indexes have been found to be associated with diabetic nephropathy risk in women but not in men [37]. This suggests that women with T2DM should focus on maintaining a healthy weight, targeting the reduction of both central and general obesity, to enhance nephroprotection. In terms of body composition, the association between the predicted body mass index (BMI) and fat mass index (FMI) with incident diabetic nephropathy differs between men and women [38]. Consistent with previous literature, our study also found that the mean BMI was significantly higher in the diabetic nephropathy group compared to the non-nephropathic group. These findings further support the notion that higher BMI levels are associated with an increased risk of diabetic nephropathy.

Furthermore, the levels of microalbumin in urine were significantly higher in the diabetic nephropathy group compared to the non-nephropathic group. This indicates that microalbuminuria can serve as a valuable biomarker for detecting and monitoring diabetic nephropathy as it reflects glomerular injury and increased glomerular permeability. Besides its role in the diagnosis of diabetic nephropathy, it is also associated with other conditions that are characterized with inflammation, including chronic obstructive pulmonary disease [39] and heart diseases [40].

Increased echointensity in sonography is associated with inflammatory conditions [41]. In fact, type 2 diabetes mellitus [42], and its complications [43], are associated with an increased burden of inflammation. In the literature, a lot of inflammatory markers and cytokines, such as the C reactive protein to albumin ratio [44], kidney injury molecule [45], platelet distribution width [46], serum uric acid [47], the monocyte/lymphocyte ratio in hemogram [48], neuregulin [49], and the uric acid/HDL cholesterol ratio [50], have been linked with diabetic nephropathy. Therefore, the association between echointensity and diabetic nephropathy in the present study was an expected result.

Effective management of diabetic nephropathy hinges on achieving and maintaining optimal glucose regulation. The glycosylated hemoglobin (HbA1C) level, widely recognized as the gold standard for assessing long-term glycemic control, plays a crucial role in minimizing complications associated with diabetes [51,52]. Notably, our study revealed higher serum glucose and HbA1C levels in the diabetic nephropathy group, providing further evidence of the link between inadequate glycemic control and the development of diabetic nephropathy. These findings underscore the critical significance of attaining and sustaining optimal glucose levels as a preventive and therapeutic measure for diabetic nephropathy.

Additionally, we found a significantly higher median serum creatinine level in the diabetic nephropathy group, indicating impaired renal function. This underscores the progressive nature of diabetic nephropathy and the potential for it to lead to end-stage renal disease.

Limitations of present study may include its retrospective design, single center nature, and relatively small study cohort. However, to the best of our knowledge, the present study is the first in the literature to investigate the evaluation of the hepatic/renal-splenic/renal echointensity ratio in diabetic nephropathy.

## 5. Conclusions

To summarize, the findings of our study shed light on the specific renal alterations in diabetic nephropathy and support the potential use of echointensity measurements as noninvasive indicators of renal damage in this patient population. The observed changes in renal echointensity, along with the absence of corresponding alterations in splenic and hepatic echointensity, highlight the kidney-specific nature of these changes. Furthermore, quantitative assessment proves to be more valuable than qualitative assessment, as it provides a more objective and reliable measure of renal echointensity and enhances our understanding of the pathophysiology and progression of diabetic nephropathy.

## Figures and Tables

**Table 1:** Demographic characteristics of the study population.

		Diabetic Nephropathic Subjects (*n* = 24)	Diabetic Non-Nephropathic Subjects (*n* = 35)	*p*
Gender	Women	10 (42%)	20 (57%)	0.24
	Men	14 (58%)	15 (43%)	0.24
Age (years)		61.7 ± 10	58.5 ± 12	0.29

**Table 2:** The data of diabetic nephropathy and non-nephropathic groups.

	Diabetic Nephropathic Subjects (*n* = 24)	Diabetic Non-Nephropathic Subjects (*n* = 35)	*p*
	Mean (±Std)	Mean (±Std)	
BMI (kg/m^2^)	32.4 ± 1	29.1 ± 0.7	0.02
Albumin (g/dL)	4.6 ± 0.4	4.7 ± 0.4	0.58
Right renal width (mm)	45 ± 5.5	44 ± 5.4	0.58
Left renal length (mm)	112 ± 10.7	110 ± 9.9	0.42
Left renal width (mm)	51 ± 6.7	49 ± 6.2	0.17
Left renal cortical thickness (mm)	14.5 ± 0.4	16.2 ± 0.4	0.006
Spleen length (mm)	103 ± 15.6	99 ± 13.3	0.27
Splenic/renal echointensity ratio %	0.60 ± 0.2	1.02 ± 0.2	<0.001
	Median (min–max)		
Microalbumin in Urine (mcg/mL)	122.5 (33–2000)	5 (4–18)	<0.001
Glucose (mg/dL)	158 (89–432)	137 (73–313)	0.046
HbA1C (%)	8.5 (7.1–12.6)	7.2 (5.2–12.2)	<0.001
AST (U/L)	18 (10–46)	18 (10–41)	0.91
ALT (U/L)	21 (11–58)	19 (10–49)	0.62
Urea (mg/dL)	35 (12–92)	35(23–58)	0.88
Creatinine (mg/dL)	0.92 (0.58–9)	0.80 (0.51–1.31)	0.01
eGFR (mL/min/1.73 m^2^)	81 (119–187)	90 (55–111)	0.13
Liver length (mm)	135 (24–56)	141(113–175)	0.85
Liver echogenicity (grade)	2 (0–3)	1 (0–2)	0.29
Right renal length (mm)	102 (88–130)	108.5 (96–133)	0.04
Right renal cortical thickness (mm)	15 (11–18)	14 (8–18)	0.06
Right renal echogenicity (grade)	0 (0–1)	0 (0–2)	0.17
Left renal echogenicity (grade)	0 (0–1)	0 (0–1)	0.06
Liver echointensity	92 (32–111)	67 (34–103)	0.40
Right renal echointensity %	59.6 (44.6–80.8)	32.7 (28.7–52)	<0.001
Left renal echointensity %	58 (41.4–83.5)	33.9 (29.1–58.7)	<0.001
Spleen echointensity	35 (31–39)	35 (31–57)	0.62
Hepatic/renal echointensity ratio %	1.43 (0.44–2.2)	1.9 (1.05–3.3)	0.025

**Table 3:** The ROC analysis results in diabetic nephropathy groups.

	Sensitivity %	Specificity %	AUC	*p*	95% CI (U-L)
Right renal echointensity > 44.15	100	97	0.99	<0.001	0.98–1
Left renal echointensity > 39.18	100	91	0.98	<0.001	0.95–1
Hepatic/renal echointensity ratio < 1.9	87	51	0.67	0.03	0.53–0.81
Splenic/renal echointensity ratio < 1.2	100	97	0.98	<0.001	0.94–1

## Data Availability

Data related to this work are available from the corresponding author on reasonable requests.
